# Lymph node status have a prognostic impact in breast cancer patients with distant metastasis

**DOI:** 10.1371/journal.pone.0182953

**Published:** 2017-08-14

**Authors:** Chuangang Tang, Pei Wang, Xiaoxin Li, Bingqing Zhao, Haochang Yang, Haifeng Yu, Changwen Li

**Affiliations:** 1 Department of Breast Surgery, Xuzhou Central Hospital, The Affiliated Xuzhou Hospital of Medical College of Southeast University, Xuzhou, China; 2 Department of Pathology, Xuzhou Central Hospital, The Affiliated Xuzhou Hospital of Medical College of Southeast University, Xuzhou, China; 3 Department of Surgery, Tianjin Second People's Hospital, Tianjin, China; 4 College of Clinical Medicine, Binzhou Medical University, Yantai, China; 5 Department of General Surgery, Tianjin First Central Hospital, Tianjin, China; University of North Carolina at Chapel Hill School of Medicine, UNITED STATES

## Abstract

**Background:**

The objective of this retrospective study was to determine whether lymph node metastasis has a prognostic impact on patients with stage IV breast cancer.

**Patients and methods:**

Seven thousand three hundred and seventy-nine patients with *de novo* stage IV breast cancer diagnosed from 2004 to 2013 were identified. Kaplan-Meier estimate method was fitted to measure overall survival and breast cancer-specific survival (BCSS). Cox proportional hazard analysis was used to evaluate the association between N stage and BCSS after controlling variables such as other patient/tumor characteristics.

**Results:**

The primary site of M1 tumors was mainly upper-outer quadrant and overlapping lesion of the breast. Patients with N1 disease had better overall survival and BCSS than did those without lymph node metastasis. The overall survival and BCSS of M1 patients with N3 disease were significantly lower than that of those with N0, N1 and N2 disease, whereas patients with N2 and N0/N1 involvement showed no significant difference with survival. Multivariate analysis showed that lymph node metastasis was an important prognostic factor for M1 patients (N1 versus N0, hazard ratio [HR] = 0.902, 95% confidence interval [CI]: 0.825–0.986, p = 0.023; N3 versus N0, HR = 1.161, 95% CI: 1.055–1.276, p = 0.002). For M1 patients, age, race, marital status, primary site, ER, PR and HER2 were the independent prognostic factors.

**Conclusions:**

The cohort study provides an insight into *de novo* stage IV breast cancer with lymph node metastasis. Our results indicated that accurate lymph node evaluation for stage IV patients is still necessary to obtain important prognostic information.

## Introduction

Breast cancer is the most common cancer in women worldwide, and 1.68 million cases are newly diagnosed annually [1]. Among them, about 6%–10% of women present with *de novo* stage IV breast cancer at diagnosis [2–4]. The median survivals of the disease with distant metastasis are ranging between 18 and 24 months [4–6]. Once metastatic disease is diagnosed, treatment is usually palliative with systemic therapy. This practice is based on prior studies, which have shown stage IV breast cancer to be an incurable disease [7–8].

The combination of primary tumor (T), regional lymph nodes (N) and metastases (M) is the cornerstone of the breast cancer staging system of the American Joint Committee on Cancer (AJCC). Based on the presence of distant metastasis, all patients are divided into two groups—staging M0 and M1 [9]. For M0 patients, lymph node metastasis is an important demarcation criterion, which is also regarded as one of the most important prognostic factors in clinical practice [9–12]. However, M1 patients are all categorized as stage IV regardless of any N status [9]. Up to date, the clinical value of N descriptors has been neglected in various cancers of M1 staging, including breast cancer. Recently, Dai et.al reported that lymph node involvement is an independent prognostic factor for M1 patients with lung cancer [13], whereas the clinical effect of lymph node status on patients with M1 breast cancer has not been studied extensively. Thus, the present study aims to identify whether accurate identification of lymph node status in M1 patients with breast cancer is of clinical value.

## Materials and methods

### Patients

The Surveillance Epidemiology and End Results (SEER) database (2004–2013) was used for the study. The National Cancer Institute’s SEER*Stat software (Version 8.2.0) was used to identify patients. All patients had a pathologically confirmed diagnosis of stage IV breast cancer according to the 6th and 7th edition of the AJCC criteria. Patients for whom breast cancer was not the first tumor were excluded. Demographics, including age, gender, race and marital status at diagnosis were retrieved. Tumor variables included location of the primary tumor, T staging, N staging, histological type, estrogen receptor (ER) status, progesterone receptor (PR) status and human epidermal growth factor receptor 2 (HER2) status. Data of HER2 status is available since 2010. Survival data were extracted at 1 mo intervals for a follow-up period between 1 mo and 120 mo.

### Statistical analysis

The data were presented as median (range) and percent values. Overall survival and breast cancer-specific survival (BCSS) were evaluated using the Kaplan-Meier method and compared using the log-rank test for all M1 patients according to N staging. Overall survival was determined from the SEER record of survival time (total number of months) and vital status. Breast cancer–specific survival (BCSS) was defined as the interval from diagnosis of M1 disease until death due to breast cancer. In addition, multivariate the Cox proportional-hazard regression model was applied to adjust for potential confounders in the survival analysis for all M1 patients, with *p*-values < 0.05 considered statistically significant. All analyses were conducted using SPSS 19.0 software (IBM, Inc., Armonk, NY).

## Results

A total of 7379 patients of M1 breast cancer were selected from the SEER database. The age of the patients ranged from 19 to 99 years, with a median age of 59 years. There were 117 male and 7262 female patients. Among them, the majority (approximately 95%) were ductal and lobular neoplasms. The primary site of M1 tumors was mainly upper-outer quadrant and overlapping lesion of the breast (26.3% and 21.8%, respectively). Table 1 showed the baseline characteristics of patients.

**Table 1:** Baseline characteristics of patients with breast cancer with M1 disease.

Characteristic	M1 patients(N = 7379)
**Median age (range), yrs**	59 (19–99)
**Age**	
≤65 yrs	5050 (68.4%)
>65 yrs	2329 (31.6%)
**Sex**	
Male	117 (1.6%)
Female	7262 (98.4%)
**Marriage**	
Yes	3558 (48.2%)
No	3821 (51.8%)
**Race**	
White	5643 (76.5%)
Black	1162 (15.7%)
Other	574 (7.8%)
**Primary location**	
Nipple	51 (0.7%)
Central portion	518 (7.0%)
Upper-outer quadrant	1938 (26.3%)
Upper-inner quadrant	455 (6.2%)
Lower-inner quadrant	309 (4.2%)
Lower-outer quadrant	439 (5.9%)
Overlapping lesion	1605 (21.8%)
Other	2064 (27.9%)
**Histological type**	
Ductal and lobular neoplasms	6977 (94.6%)
Other	402 (5.4%)
**T stage**	
T0	14 (0.2%)
T1	998 (13.5%)
T2	2649 (35.9%)
T3	1282 (17.4%)
T4	2198 (29.8%)
Tx	238 (3.2%)
**N stage**	
N0	1550 (21.0%)
N1	2682 (36.3%)
N2	1485 (20.1%)
N3	1662 (22.5%)
**ER status**	
Negative	2047 (27.7%)
Positive	5008 (67.9%)
Other	324 (4.4%)
**PR status**	
Negative	3066 (41.6%)
Positive	3934 (53.3%)
Other	379 (5.1%)
**HER2 status**	
Negative	1962 (26.6%)
Positive	725 (9.8%)
Other	4692 (63.6%)

**Fig 1:**
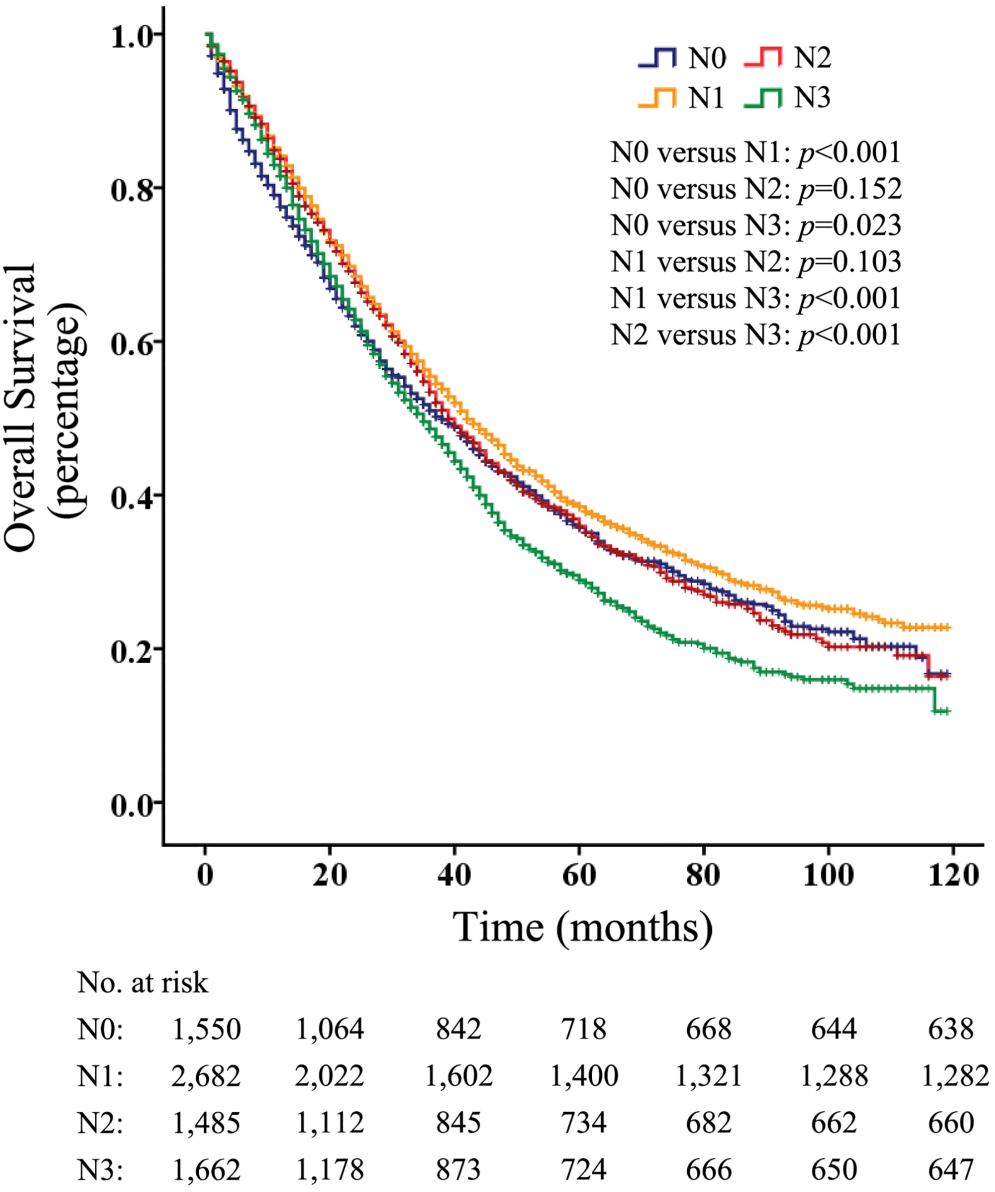
Overall survival according to N categories in patients with M1 breast cancer.

**Fig 2:**
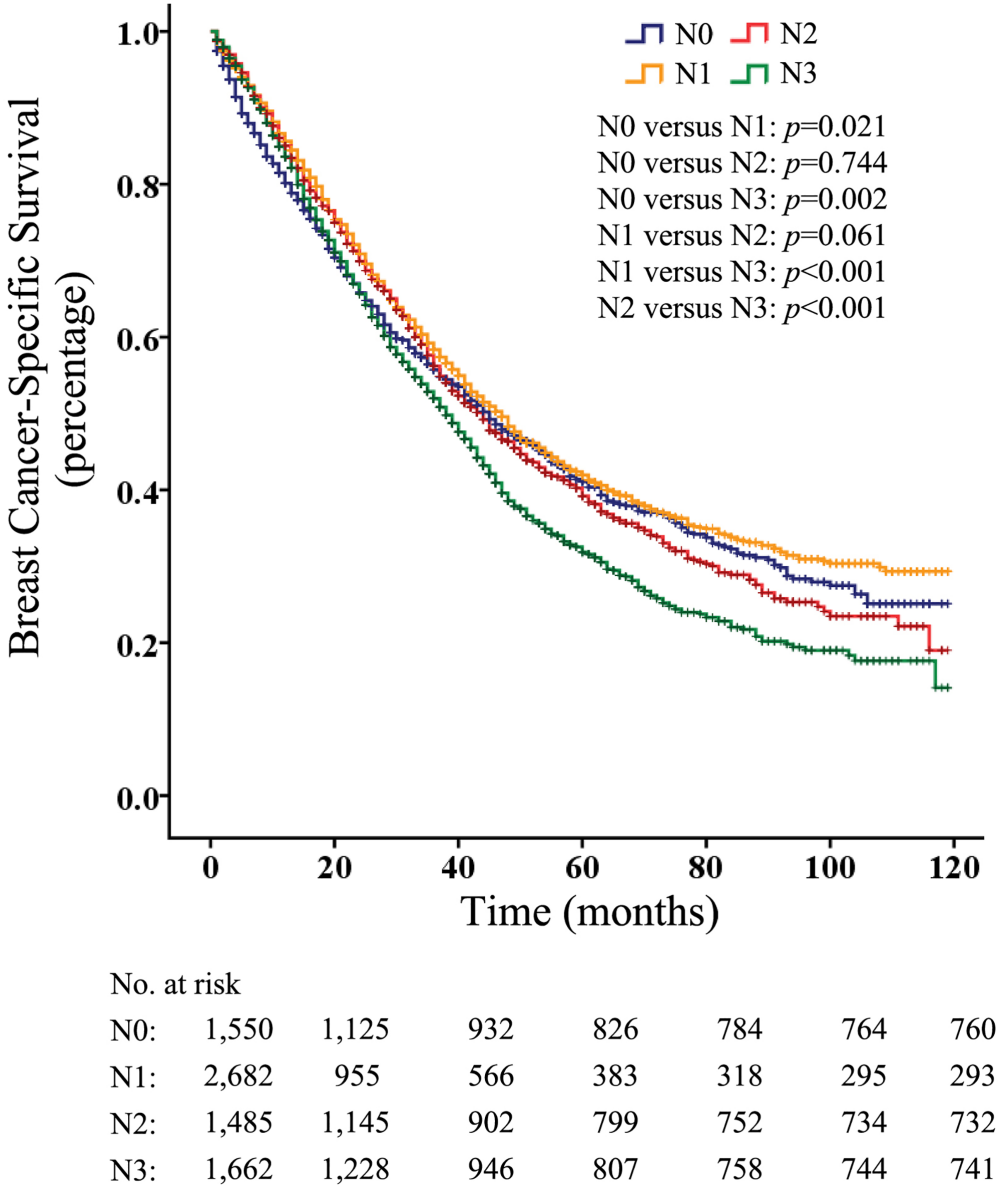
Breast cancer–specific survival according to N categories in patients with M1 Disease.

Of all the M1 patients, 1550 patients had their disease diagnosed as N0, 2682 as N1, 1485 as N2, and 1662 as N3. The survival analysis evaluating the entire cohort showed that patients with N1 disease had a better overall survival (p < 0.001) than did those without lymph node metastasis (Fig 1). In addition, the overall survival of M1 patients with N3 disease were significantly lower than that of those with N0, N1 and N2 disease (p < 0.05, p < 0.001 and p < 0.001, respectively) (Fig 1), whereas similar overall survival rates were observed between patients with N2 involvement and those with N0 or N1 involvement (p = 0.152 and p = 0.103, respectively) (Fig 1). Further, the BCSS rates of M1 patients were similar to the overall survival (Fig 2).

In the multivariate analysis, the results indicated that lymph node metastasis was an important prognostic factor for M1 patients (N1 versus N0, hazard ratio [HR] = 0.902, 95% confidence interval [CI]: 0.825–0.986, p = 0.023; N3 versus N0, HR = 1.161, 95% CI: 1.055–1.276, p = 0.002) (Table 2). In addition, the risk of death was lower for M1 patients aged ≤ 65 years than those aged > 65 years (HR = 1.417, 95% CI: 1.325–1.516, p < 0.001) (Table 2). For M1 patients, race, marital status, primary site, ER, PR and HER2 (negative vs. positive) were the independent prognostic factors (Table 2).

**Table 2:** Cox proportional hazards regression model for breast cancer–specific survival in patients with M1 disease.

Characteristic	Hazard Ratios (95% CI)	*p* value
**Age**		
≤65 yrs	1.00 (reference)	<0.001
>65 yrs	1.417 (1.325–1.516)	
**Sex**		
Male	1.00 (reference)	0.08
Female	0.806 (0.633–1.026)	
**Marriage**		
Yes	1.00 (reference)	<0.001
No	1.315 (1.233–1.403)	
**Race**		
White	1.00 (reference)	
Black	1.445 (1.328–1.572)	<0.001
Other	0.823 (0.722–0.938)	0.004
**Primary location**		
Nipple	1.00 (reference)	
Central portion	0.691 (0.473–1.009)	0.055
Upper-outer quadrant	0.770 (0.535–1.107)	0.159
Upper-inner quadrant	0.671 (0.458–0.982)	0.04
Lower-inner quadrant	0.635 (0.428–0.942)	0.024
Lower-outer quadrant	0.665 (0.454–0.976)	0.037
Overlapping lesion	0.740 (0.514–1.065)	0.105
Other	0.821 (0.571–1.180)	0.286
**Histological type**		
Ductal and lobular neoplasms	1.00 (reference)	0.02
Other	1.178 (1.026–1.351)	
**T stage**		
T0	1.00 (reference)	
T1	1.827 (0.587–5.685)	0.298
T2	1.954 (0.629–6.066)	0.246
T3	2.484 (0.799–7.719)	0.116
T4	3.239 (1.043–10.053)	0.042
Tx	2.163 (0.688–6.799)	0.186
**N stage**		
N0	1.00 (reference)	
N1	0.902 (0.825–0.986)	0.023
N2	0.983 (0.890–1.087)	0.74
N3	1.161 (1.055–1.276)	0.002
**ER status**		
Negative	1.00 (reference)	
Positive	1.571 (1.434–1.721)	<0.001
Other	1.224 (0.903–1.658)	0.193
**PR status**		
Negative	1.00 (reference)	
Positive	1.574 (1.442–1.718)	<0.001
Other	1.435 (1.080–1.907)	0.013
**HER2 status**		
Negative	1.00 (reference)	
Positive	1.915 (1.595–2.298)	<0.001
Other	1.797 (1.518–2.127)	<0.001

## Discussion

The TNM classification system attempts to account for most basic parameters of cancer, and it has utility for determining the extent of disease, providing guidance for treatment planning and predicting the outcome. As the single most important prognostic factor in breast cancer [14–15], the nodal status in M0 patients has attracted much more attention than that in M1 patients. Remarkably, the results from the current study showed that lymph node metastasis was an important prognostic factor for patients with M1 breast cancer.

In addition, patients without lymph node metastasis had worse overall survival and BCSS than did those with N1 disease, which was confusing indeed. For one hand, T stage has not been taken into consideration. For example, patients with T1N1 should have better survival than those with T3N0. For another, the invasion of tumor cell into lymph nodes can activate an antitumor immune response, which may benefit patients with lymph node metastasis[16].

Patients with N2 and N0/N1 involvement showed no significant difference with survival (P > 0.05). We speculate that the abnormality may be related to the site of metastasis, such as visceral metastases, bone metastases and brain metastases. Several studies have reported a range of prognostic factors for women with metastatic breast cancer including factors such as age at diagnosis, ER, PR, HER2 and site of metastases [5,17]. The current study showed that for M1 patients, age, race, marital status and primary site were the independent prognostic factors. Thus, further studies or more clinical data are required to evaluate the impact of the site of metastasis on survival in M1 patients with different N stages. Additionally, several studies have reported improvement in survival of women with metastatic breast cancer, such as partial or total mastectomy [18–19]. Hence, different clinical treatments may have influence on survival in M1 patients.

Prognostic factors combining clinical and laboratory variables with physician’s estimates have been developed in recent years [20]. However, in this study, we just selected patients from the SEER database to analyze the prognostic factors. It is necessary for us to include more detailed information using our own patient database to verify the results.

In conclusion, accurate lymph node staging is utilized mainly to estimate prognosis, and it also contributes to determining treatment strategies. Our results supported that the prognostic value of lymph node staging extends even to M1 patients and indicated that accurate lymph node evaluation for M1 patients is still necessary to obtain important prognostic information.

## Supporting information

S1 File Original data of each case from SEER database. (XLSX)
